# Social phobia following maprotiline: the first case report

**DOI:** 10.1186/1757-1626-2-9340

**Published:** 2009-12-17

**Authors:** Seyed Hamzeh Hosseini, Ebrahim Salehifar

**Affiliations:** 1 Department of Psychiatry, Faculty of Medicine, Mazandaran University of Medical Sciences, Sari, Mazandaran, Iran; 2 Department of Clinical Pharmacy, Faculty of Pharmacy, Mazandaran University of Medical Sciences, Sari, Mazandaran, Iran

## Abstract

**Introduction:**

It has long been recognized that anxiety symptoms and syndromes may be caused by medication and/or substance of abuse. The aim of this report is to present a patient who experienced social phobia following maprotiline, an adverse drug reaction which has not been reported previously with this agent.

**Case presentation:**

A 27 year-old male patient was suffering from dysthymia for 6 years. He received different kinds of pharmacotherapy including TCAs, SSRIs, MAOIs. Due to his poor response, augmenting therapy with liothyronine and lithium and also cognitive therapy have been tried for him. Since he did not respond well to these treatments, maprotiline was administered for him 50 mg daily. His psychological problems improved with maprotiline, however he experienced social phobia that has not experienced yet.

**Conclusion:**

This could underlie the precipitation of social phobia after maprotiline. However there is a need for further studies.

## Introduction

It has long been recognized that anxiety symptoms and syndromes may be caused by medications and/or substance abuse. Many medications are capable of engendering anxiety disorder including anesthetics, analgesics, antidepressants (Tricyclics, SSRIs, bupropion), antihistamines, antimicrobials, bronchodilators, calcium-blocking agents, cholinergic-blocking agents, digitalis, estrogens, ethosuximide, hydralazine, insulin, levodopa, muscle relaxants, narcoleptics, non-steroidal anti inflammatory drugs, procaine, procarbazine, sedatives, steroids, sympathomimetics, theophylline and thyroid preparations. In addition, withdrawal from CNS depressant drugs, aspirin intolerance, drug intoxication and caffeinism also have been proposed as the causes for social phobia [1].

Maprotiline is a tetra cyclic antidepressant agent which has been approved for the treatment of depression and anxiety associated with depression [2]. The aim of this report is to present a patient who experienced social phobia following maprotiline administration, an adverse drug reaction which has not been reported yet with this agent. He complained from low energy on fatigue, low self steam, feeling of hopelessness and poor concentration. These symptoms were continuously. He has never been with out the symptoms more than 2 month at a time.

## Case Presentation

A 27 year-old male patient (was suffering from dysthymia for 6 years). He did not have any anxiety disorder, mood disorder, substance use disorder, and personality disorder ago. However, he had a family history of mood disorder, in one of his first degree relatives his brother was sufferings from dysthymia.

He has been taken various treatments including pharmacotherapy and psychotherapy and biological therapy. He has adequate courses of amitriptyline, nortriptyline, clomipramine, fluoxetine, trazodone, sertraline, and tranylcypromine augmented by liothyronine, lithium and cognitive therapy for six month. In spite of all of these therapies, his condition did not improve.

Finally, he received maprotiline 50 mg daily. His condition improved by maprotiline, significantly. Wide range of social phobia symptoms including flushing, tachycardia and tremulousness occurred when he was talking to other peoples. In other words he had a marked and persistent fear of more social or performance situation in which he is exposed to unfamilies people or to possible scrutiny by other. These symptoms were disappeared following dosage reduction to 25 mg maprotiline daily.

## Discussion

As well as we know, there is not any report regarding the social phobia following maprotiline administration. Maprotiline is a tetra cyclic antidepressant drug with actions and uses similar to those of tricyclic antidepressants. It is a selective inhibitor of the neither reuptake of nor epinephrine [3].

Adverse effects with maprotiline are broadly similar to those with tricyclic antidepressants, but antimuscarinic effects are less frequent. Skin rashes seem more common with maprotiline than with tricyclic antidepressants. Seizures have occurred in patients with no prior history of such disorders as well as in those with a history of epilepsy and the risk are increased if high doses of maprotiline are employed. It should not be used in patients with epilepsy or a lowered seizure threshold [4].

It is postulated that nor epinephrine may has an important role in the path physiology of social phobia [5].

Patients with performance phobias may release more norepinephrin or epinephrine, both centrally and peripherally, than do non phobic person or such patients may be sensitive to a normal level of adrenergic stimulation[6].

The locus ceruleus area in the brainstem is the major source of nor epinephrine, and its stimulation produces arousal and symptoms of anxiety. Over activity of noradrenergic neurons may underlie some cases of anxiety, and elevated concentrations of nor epinephrine and its major metabolite, 3-methoxy-4-hydroxy phenyl glycol (MHPG), have been found in the plasma and cerebrospinal fluid of anxious patients, Administration of yohimbine, an antagonist of the presynaptic α-2 adrenergic auto receptor, increases nor epinephrine release in the locus ceruleus and produces anxiety in humans. Drugs that decrease noradrenergic function can possess anxiolytic effects. Clonidin, a presynaptic α-2 adrenergic agonist that decreases noradrenergic activity, reduces anxiety symptoms and has also been effective in the treatment of alcohol and opiate withdrawal syndromes characterized by symptoms of anxiety [7]. Facilitation of gamma-amino butyric acid (GABA) by benzodiazepines also reduces noradrenergic activity. (Considering the pharmacodynamic action of maprotiline and the above mentioned evidences regarding the role of nor epinephrine in the path physiology of social phobia, it would be reasonable to explain this adverse reaction to the over activity of the sympathetic pathways).

However School avoidance, social phobia and separation anxiety have been reported as potential consequences of neuroleptic treatment of patients with Tourette's disorder.

Mikkelsen and colleagues reported a series of case reports including 15 children and adults who experienced anxiety and work avoidance with haloperidol happened weeks to months after beginning of treatment. The mean doses were 2.5 mg/day. The phobic syndrome was not associated with akathisia, and disappeared completely in all patients with the discontinuation of the drug [8]. Pimozide has also been reported to produce school phobia and separation anxiety [9].

## Conclusion

Clinicians should be alert to social phobia as a possible adverse reaction of maprotiline, and on the basis of this reported case, we suggest that this reaction could be treated successfully with dosage reduction.

## Patients' Perspective

I am suffering from low energy on fatigue, low self steam, feeling of hopelessness and poor concentration continuously. When I am talking to other peoples, I feel flushing and palpitation. In other words I have a marked and persistent fear of more social or performance situation in which I am exposed to unfamiliar people.

## Abbreviations

**SSRIs**: selective serotonin reuptake inhibitor; **CNS**: central nervous system; **MHPG**: 3-methoxy-4-hydroxy phenyl glycol; **GABA**: gamma-amino butyric acid; **TCAs**: tricyclic antidepressants.

## Consent

Written informed consent was obtained from the patient for publication of this case report and accompanying images. A copy of the written consent is available for review by the Editor-in-Chief of this journal.

## Competing interests

The authors declare that they have no competing interests.

## Authors' contributions

SHH was attending, corresponding author and the major contributor in writing the manuscript. ES was pharmacologist and as a contributor in writing the manuscript too.
